# A multi-center phase II study and biomarker analysis of combined cetuximab and modified FOLFIRI as second-line treatment in patients with metastatic gastric cancer

**DOI:** 10.1186/s12885-017-3174-z

**Published:** 2017-03-14

**Authors:** Xin Liu, Weijian Guo, Wen Zhang, Jiliang Yin, Jun Zhang, Xiaodong Zhu, Tianshu Liu, Zhiyu Chen, Biyun Wang, Jianhua Chang, Fangfang Lv, Xiaonan Hong, Huijie Wang, Jialei Wang, Xinmin Zhao, Xianghua Wu, Jin Li

**Affiliations:** 1 Department of Medical Oncology, Fudan University Shanghai Cancer Center, 270 Dong-An Road, Shanghai, 200032 China; 2 Department of Oncology, Ruijin Hospital of Shanghai Jiaotong University School of Medicine, Shanghai, 200025 China; 3 Department of Medical Oncology, Zhongshan Hospital of Fudan University, Shanghai, 200032 China

**Keywords:** Cetuximab, FOLFIRI, Gastric cancer, Biomarker

## Abstract

**Background:**

To evaluate the efficacy of cetuximab combined with modified FOLFIRI (mFOLFIRI) as a second-line treatment in metastatic gastric cancer patients and to identify potential biomarkers of clinical outcomes.

**Methods:**

All 61 patients received an initial intravenous (IV) dose of cetuximab (400 mg/m^2^) and weekly doses (250 mg/m^2^) thereafter, starting on day 1. On day 2 of each 14-day period, patients received IV irinotecan (180 mg/m^2^), leucovorin (200 mg/m^2^), and an IV bolus dose of 5-FU (400 mg/m^2^) followed by a continuous infusion of 5-FU (2400 mg/m^2^) for 46 h. The primary endpoint was time-to-progression (TTP).

**Results:**

The response rate (RR) was 33.3% among 54 evaluable patients. In the intention-to-treat analysis, median TTP was 4.6 months (95% confidential interval [CI]: 3.6-5.6 months) and median overall survival (OS) was 8.6 months (95% CI: 7.3-9.9 months). In univariate analyses, plasma vascular endothelial growth factor (VEGF) levels were correlated with clinical outcome. In patients with low (≤12.6 pg/ml) and high (>12.6 pg/ml) baseline plasma VEGF levels, RR values were 55.0% and 5.3%, respectively (*P* = 0.001); median TTP values were 6.9 months and 2.8 months, respectively (P = 0.0005); and median OS values were 12 months and 5 months, respectively (*P* <0.0001). None of these patients exhibited KRAS, BRAF, or PIK3CA mutations.

**Conclusions:**

Combination therapy comprising cetuximab and mFOLFIRI was well tolerated and active as a second-line treatment for patients with metastatic gastric cancer. Patients with low baseline plasma VEGF levels were associated with better clinical outcomes.

**Trial registration:**

ClinicalTrials.gov. NCT00699881. Registered 17 June 2008 (retrospectively registered)

## Background

Metastatic gastric cancer (MGC), an incurable disease with a poor prognosis, is marked by a short median overall survival (OS) time. Chemotherapy comprising fluoropyrimidine and platinum (combined with trastuzumab in HER-2 positive patients) has been considered as standard therapeutic regimen in the first-line setting [1–3].

Almost all of the MGC patients experienced disease progression afte first-line treatment. Salvage chemotherapy (SLC), as second-line treatment, has been shown to significantly improve survival when added to best supportive care (BSC). A large Korean study randomized patients with MGC with one or two prior chemotherapy regimens (70% one prior therapy) to SLC (either docetaxel or irinotecan) plus BSC or BSC alone, and found that median OS was prolonged in the SLC arm (5.3 vs. 3.8 months), with no median OS difference between docetaxel and irinotecan [4]. A Japanese phase III study (WJOG4007) compared treatment with paclitaxel and irinotecan in patients with MGC refractory to treatment with fluoropyrimidine plus platinum. This study reported no significant difference between paclitaxel and irinotecan for OS [5]. Thus, both irinotecan and taxanes are reasonable second-line treatment options for MGC. The RAINBOW study showed ramucirumab (a VEGFR-2 antagonist) could increase median OS when combined with paclitaxel in second-line treatment for patients with MGC [6].

However, the efficacy of second-line chemotherapy for MGC is still very limited. It’s urgently needed to improve the prognosis of these patients. The combination of cetuximab (an EGFR antagonist) and irinotecan has been widely used in the second or third-line treatment of metastatic colorectal cancer (mCRC) patients [7, 8]. The BOND study found that cetuximab may circumvent irinotecan resistance in patients with irinotecan refractory tumors [9]. At the time of our study design, some phase II trials assessed cetuximab combined with chemotherapy in the first-line or second-line treatment of gastric cancer [10, 11]. Since irinotecan is one of the major drugs used in the second-line treatment for MGC, and enlightened by the striking synergistic effects from the irinotecan-cetuximab combination in mCRC, we presumed that irinotecan-cetuximab combination may improve the efficacy in second-line treatment for MGC. Then we did some preclinical studies to explore whether cetuximab could enhance the activities of irinotecan on gastric cancer cell lines, and the results showed significant potentiation of antiproliferative, apoptosis and G2/M phase arrest effects in response to the addition of cetuximab to irinotecan in GC cell lines via the downregulation of the EGFR pathway upregulated by irinotecan [12].

Therefore, this phase II clinical trial (NCT00699881) was designed to evaluate the safety and efficacy of cetuximab combined with modified FOLFIRI (mFOLFIRI) in patients with MGC who failed to first-line chemotherapy. Plasma protein levels of VEGF and EGF, gene mutations of KRAS, BRAF and PIK3CA, and expression of P27, phosphorylated EGFR and AKT in tumor tissues were also investigated for their potential roles as biomarkers of clinical outcomes.

## Methods

### Patient eligibility

This open-label, single-arm, multicenter, phase II study included patients who met the following eligibility criteria: aged between 18 and 70 years; histologically confirmed metastatic or locally advanced gastric adenocarcinoma with at least one measurable lesion in a non-irradiated area; one prior chemotherapy regimen (except adjuvant chemotherapy); Eastern Cooperative Oncology Group (ECOG) performance status (PS) of 0 or 1; adequate organ function (bone marrow function: neutrophil count [ANC] ≥2.0 × 10^9^/L, platelet count [PLT] ≥80 × 10^9^/L; liver function: serum bilirubin and serum transaminase levels ≤1.5 × ULN [upper limit of normal]; renal function: serum creatinine ≤1.0 × ULN). The following criteria were applied for patient exclusion from the study: patients who received cetuximab or irinotecan as a first-line chemotherapy; pregnant or breast-feeding or were of child-bearing potential without using adequate contraception; had any other current or prior malignancy (with the exception of excised cervical carcinoma in situ or squamous cell skin carcinoma treated by surgery only); had central nervous system metastases; had severe or uncontrolled medical conditions (e.g., impaired heart and lung function, diabetes, active infections, or liver disease).

This study was approved by the Fudan University Shanghai Cancer Center Institutional Review Board and conducted according to the Declaration of Helsinki. All patients provided written informed consent prior to participation in this study.

### Treatment and assessment

Cetuximab was administered at an initial dose of 400 mg/m^2^, followed by weekly infusions (250 mg/m^2^). On day 2 of each 14-day period, patients received IV irinotecan (180 mg/m^2^) and LV 200 mg/m^2^ and then 5-FU (400 mg/m^2^) IV bolus followed by a continuous infusion of 5-FU (2400 mg/m^2^) for 46 h. Treatment was continued until development of progressive disease (PD), occurrence of unacceptable toxic effects, or withdrawal of patient consent. Dose reductions and/or administration delays were applied in cases of febrile neutropenia, grade 4 myelosuppression, or grade 3/4 non-hematological toxic effects. In cases where chemotherapy was discontinued due to its toxicity, patients were allowed to continue with cetuximab. A special dose reduction scheme was specified for skin-related toxic effects.

Response evaluation was performed according to the Response Evaluation Criteria in Solid Tumors (RECIST) every eight weeks during treatment period and every 3 months after treatment was discontinued. Complete responses (CR) or partial responses (PR) were confirmed with CT scans performed at least 4 weeks apart. Adverse events (AEs) including rash were evaluated according to the National Cancer Institute Common Terminology Criteria for Adverse Events (version 3.0).

### Biomarker analyses

#### Plasma EGF and VEGF level analysis

Venous blood for cytokine assessment was drawn into an ethylenediaminetetraacetic acid (EDTA) anticoagulant tube immediately prior to the first drug infusion. Each venous blood sample was immediately centrifuged for 10 min at 4,000 rpm and the plasma was stored at -80 °C for subsequent assay of vascular endothelial growth factor (VEGF) and endothelial growth factor (EGF) levels by enzyme-linked immunosorbent assay (ELISA) according to the instructions provided by the manufacturer (Invitrogen, US). All samples were assayed in duplicate.

#### Mutation analysis

Mutation analysis of KRAS, BRAF, and PIK3CA genes was performed by extraction of genomic DNA from formalin-fixed, paraffin-embedded tissue slides or sections using the QIAamp DNA Mini Kit (Qiagen, Germany). DNA was amplified using oligonucleotide primers specific for human KRAS (exons 12 and 13), BRAF (V600E) and PIK3CA (exons 9 and 20) genes and then screened with pyrosequencing.

#### Protein expression analysis by immunohistochemical staining

Immunohistochemical (IHC) staining of tumor samples was carried out to assess the expression of phosphorylated EGF receptor (pEGFR), and EGFR downstream molecules, such as phosphorylated AKT (pAKT), P27 and m-TOR. PTEN expression was also analysed, which located in upstream of PI3K/AKT. Positive staining was defined as staining above background level in ≥10% of cancer cells.

### Statistical considerations

The primary endpoint was time-to-progression (TTP). This study was designed to test the hypothesis that a median TTP value of 4.0 months (H_1_) obtained in this study is significantly different from the value of 2.5 months (H_0_), which represents the median TTP of FOLFIRI as the second-line treatment for gastric cancer. Sample size was determined following Gehan’s two-stage phase II optimal trial design. Fifteen patients were enrolled in the first stage. If TTP ≥ 4 months was observed in five or more patients, the study proceeded to the second stage where an additional 31 patients were enrolled. Assuming a 20% drop-out rate, a total of 55 patients were required for this study.

The secondary endpoints of the study included the RR, OS, AEs, and potential biomarkers. Survival curves were generated using the Kaplan-Meier method and comparisons of TTP and OS between groups were performed by log-rank tests. Safety analysis was performed for the safety population, which consisted of all patients who received at least one dose of cetuximab. As an exploratory endpoint, activating mutations of the KRAS, BRAF, and PIK3CA genes, expression of pEGFR, pAKT, P27, mTOR and PTEN in tumor samples, plasma protein level of VEGF, EGF, and their association with efficacy and prognosis were also analyzed. A receiver operating characteristic (ROC) curve analysis was used for selection of a cut-off point for the ligand level, which was defined as the ligand level with the highest sensitivity and specificity for the response. Statistical analysis of the correlation between biomarker status and RR was carried out using a Pearson’s *χ*2 test or Fisher’s Exact test.

TTP and OS were analyzed in the intent-to-treat (ITT) population. TTP was calculated from the day of the first infusion to the date of documented disease progression or last contact. Patients who had not progressed at the time of the final analysis were censored at the date of their last tumor assessment. OS was calculated from the day of the first infusion to death. Patients alive at the final survival analysis were censored using the last contact date. Statistical analyses were performed using SPSS software (version 12.0; SPSS, Chicago, IL, USA).

## Results

### Patient disposition

Between May 2008 and November 2009, 61 patients with metastatic gastric cancer were enrolled into the study from three participating hospitals. All 61 patients were evaluated for safety and survival, and 54 were assessable for response. Seven patients were not assessable for response due to discontinuation without tumor assessment within the first cycle of treatment as a result of obstructive jaundice (*n* = 1), febrile neutropenia (*n* = 3), and intestinal obstruction (*n* = 3). At the time of data cut-off at the end of December 2010, all patients had discontinued treatment.

### Patient characteristics

Of the 61 patients enrolled, 56% were male (*n* = 34) and 44% were female (*n* = 27), with a median age of 52 years (range 26-69). All treated patients had an ECOG PS of 0 or 1 (PS 0: 28%; PS 1: 72%). The primary tumor was located at the gastroesophageal junction (GEJ) in 23% of the patients and at other parts of the stomach in 77% of the patients. Prior surgery of the primary tumor had been performed in 66% of the patients. All patients presented with metastatic disease. The predominant metastatic sites were abdominal lymph nodes (56%), liver (44%), and lung (18%). First-line chemotherapy regimens used in the study population were as follows: 56% of the patients received ECF (epirubicin, cisplatin, 5-FU) and its variants (fluorouracil replaced by capecitabine and/or cisplatin by oxaliplatin), 21% received fluoropyrimidine plus oxaliplatin, 21% received fluoropyrimidine plus docetaxel or paclitaxel, and 2% received capecitabine monotherapy (Table 1).

**Table 1:** Patient characteristics

Demographic or Clinical Characteristic	Number of Patients (*n* = 61)	Percentage (%)
Gender		
Male	34	56
Female	27	44
Age, years		
≤ 59	44	72
> 59	17	28
ECOG PS		
0	17	28
1	44	72
Primary tumor site		
Stomach	47	77
Gastroesophageal junction	14	23
Prior surgery of primary tumor		
Yes	40	66
No	21	34
Sites of metastatic disease		
Abdominal lymph node	34	56
Liver	27	44
Primary	14	23
Lung	11	18
Ovarian	10	16
Distant lymph node	10	16
Peritoneum	9	15
Others	11	18
First-line chemotherapy		
5-FU/capecitabine + oxaliplatin	13	21
ECF/EOF/EOX	34	56
5-FU plus TXT/PTX	13	21
Capecitabine	1	2

Abbreviations: *ECOG* Eastern Cooperative Oncology Group, *PS* performance status, *5-FU* 5- fluorouracil, *ECF* epirubicin, cisplatin, 5-FU, *EOF* epirubicin, oxaliplatin, 5-FU, *EOX* epirubicin, capecitabine, oxaliplatin, *TXT*, docetaxel, *PTX*, paclitaxel

### Efficacy

The best overall responses are listed in Table 2. Fifty four patients were evaluable for response including one complete remission and 17 partial responses, resulting in a RR of 33.3% (18/54) patients (95% CI, 20.7% to 45.9%). Stable disease (SD) was observed in 50% (27/54) of patients (95% CI 43.3%–56.7%) and PD in 16.7% (9/54) of patients (95% CI 6.8%–26.6%). The DCR (CR + PR + SD) was 83.3% (95% CI 73.4%–93.2%).

**Table 2:** Overall responses

	Number	Percentage (%)
Assessable patients	54	100
Overall response	18	33.3
CR	1	1.9
PR	17	31.5
SD	27	50.0
PD	9	16.7
DCR (CR + PR + SD)	45	83.3

Abbreviations: *CR* complete response, *PR* partial response, *SD* stable disease, *PD* progressive disease, *DCR* disease control rate

**Fig. 1:**
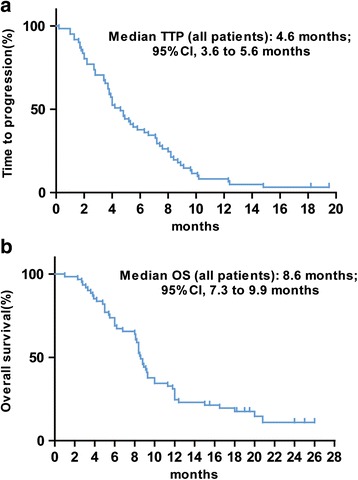
Kaplan–Meier estimates of (**a**) time-to-progression (TTP) and (**b**) overall survival (OS) among patients with metastatic gastric cancer treated with cetuximab, irinotecan, folinic acid and 5-fluorouracil (FOLFIRI)

The median follow-up time was 16 months. At the time of analysis, 97% (59/61) of enrolled patients presented with progressive disease and 15% (9/61) remained alive. In the ITT population, median TTP was 4.6 months (95% CI, 3.6 to 5.6 months; Fig. 1a) and the median OS was 8.6 months (95% CI, 7.3-9.9 months; Fig. 1b). In an analysis of TTP and OS in relation to tumor response, patients with a CR or PR had longer TTP times (median: 8.6 months vs. 4.0 months, *P* = 0.006) and OS times (median: 13.7 months vs. 7.0 months, *P* = 0.0016) compared with patients with SD or PD.

### Safety

The median number of infusions of cetuximab was 18.0 (1–48), while the median number of cycles of FOLFIRI was 8.0 (0–19). All 61 patients were evaluated for toxicity. Treatment was generally well tolerated and the major toxicity observed was hematological. Grades 3/4 neutropenia, anemia and thrombocytopenia occurred in 52.5%, 29.5%, and 8.2% of patients, respectively. Febrile neutropenia was recorded in 13.1% of patients. Overall, non-hematological toxicities were moderate and severe episodes were rare. The most common grades 3/4 non-hematological toxicities were nausea (8.2%), vomiting (6.6%), asthenia (4.9%), infection (4.9%), stomatitis (1.6%), and diarrhea (6.6%). Cetuximab-related grade 3 hypersensivity reaction was reported in one patient (1.6%). All grades of acne-like rash occurred in 70.8% (51/61) of patients and grades 3/4 toxicities were observed in 9.8% (6/61) of patients (Table 3). No other serious adverse events were observed.

**Table 3:** Grade 3 or 4 Adverse Events (National Cancer Institute Common Toxicity Criteria, Version 3.0)

	Number (*n* = 61)	Percentage (%)
Hematological toxicity		
Neutropenia	32	52.5
Febrile neutropenia	8	13.1
Anemia	18	29.5
Thrombocytopenia	5	8.2
Non-hematological toxicity		
Nausea	5	8.2
Vomiting	4	6.6
Stomatitis	1	1.6
Diarrhea	4	6.6
Infection	3	4.9
Asthenia	3	4.9
Intestinal obstruction	4	6.6
Elevated aminotransferase	1	1.6
Allergic reaction	1	1.6
Rash	6	9.8

### Biomarker analyses

#### Plasma protein level analysis

**Fig. 2:**
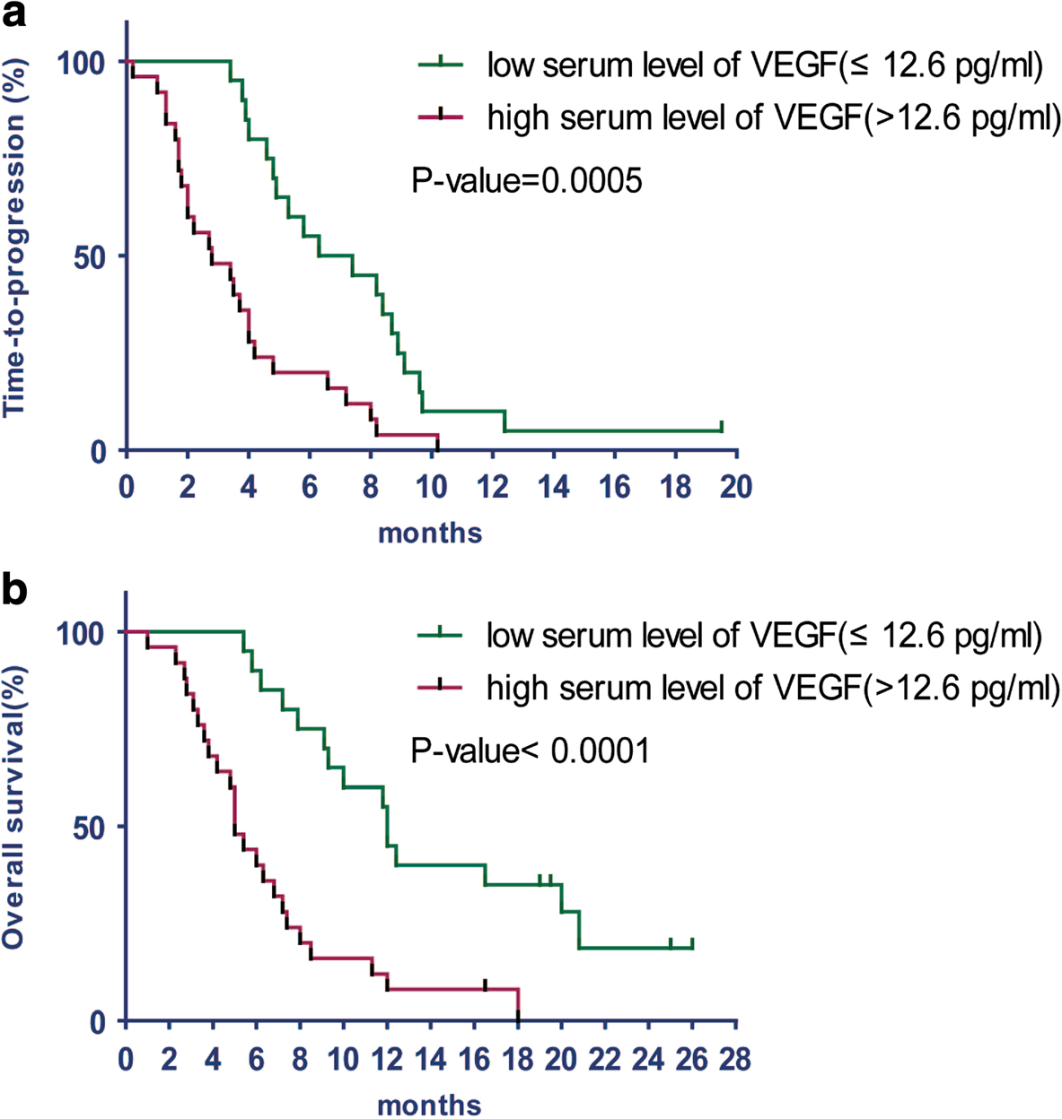
Kaplan–Meier curves of time-to-progression (**a**) and overall survival (**b**) according to serum protein level of vascular endothelial growth factor (VEGF). P-value by log-rank test

A ROC curve analysis showed that the cut-off point for the VEGF level was 12.6 pg/ml. In patients with low (≤12.6 pg/ml) and high (>12.6 pg/ml) baseline plasma VEGF levels, RR values were 55.0 and 5.3%, respectively (*P* = 0.001); median TTP values were 6.9 months and 2.8 months, respectively (*P* = 0.0005); and median OS values were 12 months and 5 months, respectively (*P* <0.0001) (Fig. 2). Baseline plasma EGF levels did not correlate with any of the clinical outcomes (Table 4).

**Table 4:** Univariate analyses of biomarker and treatment outcomes

		RR (%)	*P*-value	Median TTP (mo)	*P*-value	Median OS (mo)	*P*-value
Tumor expression (IHC)							
pEGFR	negative	32.4	0.79	5.3	0.50	7.8	0.52
	positive	28.6		4.3		9.1	
pAKT	negative	29.6	0.78	5.2	0.50	8.1	0.39
	positive	33.3		4.0		9.1	
P27	negative	23.1	0.22	4.9	0.25	7.3	0.33
	positive	40.9		5.6		9.2	
	positive	34.3		5.1		9.2	
PTEN	negative	31.1	0.56	4.4	0.28	8.2	0.39
	positive	35.2		4.9		9.0	
Serum protein level (ELISA) (pg/ml)							
VEGF	≤12.6	55.0	**0.001**	6.9	0.0005	12	**<0.0001**
	>12.6	5.3		2.8		5	
EGF	≤0.70	31.8	1.00	4.7	0.61	8.3	0.58
	>0.70	29.4		4.0		8.9	

*RR* response rate, *TTP* time-to-progression, *OS* overall survival, *mo* months, *IHC* immunohistochemistry, *PEGFR* phosphorylated epidermal growth factor receptor, *PAKT* phosphorylated AKT, *ELISA* enzyme-linked immunosorbent assay, *VEGF* vascular endothelial growth factor, *EGF* epidermal growth factor, *mTOR* mammalian target of rapamycin, *PTEN* phosphatase and tensin homolog deleted on chromosome ten. *P* < 0.05 are significant and marked in bold

#### Mutational analysis

Forty DNA samples were evaluable for gene mutation analysis. None of the patients in this study exhibited KRAS, BRAF or PIK3CA mutations.

#### Protein expression analysis

Fifty-one tumor samples were available for protein expression analysis. pEGFR expression was detected in 27.5% (14/51) of patients. In pEGFR-negative and pEGFR-positive patients, RR were 32.4 and 28.6%, respectively (*P* = 0.791); median TTP were 5.3 months and 4.3 months, respectively (*P* = 0.503); and median OS were 7.8 months and 9.1 months, respectively (*P* = 0.520). pAKT expression was detected in 47.1% (24/51) of patients (47.1%). In pAKT-negative and pEGFR-positive patients, RR were 29.6% and 33.3%, respectively (*P* = 0.776); median TTP were 5.2 months and 4.0 months, respectively (*P* = 0.497); and median OS were 8.1 months and 9.1 months, respectively (*P* = 0.394). We have also detected protein expression of P27 and mTOR in the tumors, which located in EGFR downstream signally pathways and protein expression of PTEN, which located in upstream of PI3K/AKT. However, no correlations were identified among P27, m-TOR and PTEN expression and RR, median TTP or OS (Table 4).

## Discussion

This phase II study was conducted to assess the efficacy and safety of cetuximab combined with mFOLFIRI as a second-line therapy in patients with metastatic gastric cancer following the failure of first-line chemotherapy. The median TTP observed in this study was 4.6 months, which exceeded the pre-specified criteria of 4 months, with a RR of 33.3%, a DCR of 83.3% and a median OS of 8.6 months. Treatment was generally well tolerated and the predominant grade 3/4 treatment-related toxic effects were neutropenia (52.5%), anemia (29.5%), and thrombocytopenia (8.2%). It seems that the median TTP observed in our study was better than in previously reported studies. In WJOG4007 study, median PFS was 3.6 months in the paclitaxel group and 2.3 months in the irinotecan group for the second-line treatment of MGC [5]. Moreover, the median TTP in our study was similar with that of ramucirumab plus paclitaxel in RAINBOW study (median PFS was 4.4 months), which was the only successfully developed target drug combined with chemotherapy in second-line setting with the best effects [6]. So the preliminary results of our study are exciting.

Two randomised phase 3 trials assessed anti-EGFR antibodies in the first-line setting of MGC. In EXPAND trial, the patients were randomly assigned to receive chemotherapy (capecitabine plus cisplatin) or chemotherapy combined with cetuximab. The results showed mPFS was not prolonged with the addition of cetuximab to chemotherapy (5.6 months for chemotherapy alone vs 4.4 months for chemotherapy plus cetuximab) [13]. In REAL3 trial, the patients were randomly assigned to receive chemotherapy (epirubicin, oxaliplatin, and capecitabine) or chemotherapy combined with panitumumab. The results showed the addition of panitumumab to chemotherapy was associated with inferior OS (median OS: 11.3 months vs 8.8 months for chemotherapy alone and panitumumab plus chemotherapy, respectively) [14].

However, the failure of these trials may due to several reasons. Firstly, evidence in the setting of colorectal cancer suggests that oxaliplatin and capecitabine may be suboptimum partners of anti-EGFR antibodies. Preclinical studies suggest that greater synergy might exist between cetuximab and irinotecan than with oxaliplatin. Oxaliplatin was found to activate SRC in colon cancer cells by ROS-dependent pathway, which leads to the activation of EGFR signaling and decreasing of the effects of cetuximab [15]. In clinical studies, cetuximab could increase the effects of irinotecan contained regimen for patients with mCRC. However, cetuximab combined with oxaliplatin had inconsistent results in mCRC. The COIN study showed addition of cetuximab to FOLFOX or XELOX could not improve PFS and OS even in patients with KRAS wild-type untreated mCRC. However, subgroup analysis showed cetuximab could not improve PFS of patients treated with oxaliplatin plus capecitabine, while improved PFS with cetuximab was noted in individuals treated with FOLFOX [16]. The NORDIC VII study showed the effect of cetuximab was disappointing with regard to PFS and OS when added to FLOX in which oxaliplatin combined with bolus 5Fu [17]. The CALGB/SWOG 80405 study showed that the effect of cetuximab combined with FOLFOX was comparable with that of cetuximab combined with FOLFIRI [18]. The TAILOR study showed that cetuximab plus FOLFOX significantly improved PFS in the first-line treatment of patients with RAS wild-type mCRC compared with FOLFOX alone. These studies suggested that the effect of cetuximab combined with oxaliplatin contained regimen might depend on the usage of fluoropyrimidine: cetuximab might improve the effect of oxaliplatin when combined with civ 5-Fu, but couldn’t when combined with bolus 5-Fu or capecitabine. EGFR antagonists were combined with capecitabine and platinum in both EXPAND and REAL3 trials, the failure of which may attribute to the drug interactions.

Secondly, cetuximab exerts best effect when it’s used in second or third-line setting of mCRC, which has poorer prognosis. It’s harder to improve the outcome of first-line treatment because of the better efficacy compared with the salvage treatment. In EPIC study, cetuximab added to irinotecan significantly improved PFS (median, 4.0 v 2.6 months; *P* = .0001) for the second-line therapy of mCRC [7]. However, in CRYSTAL study, the improvement of median PFS with cetuximab was less conspicuous (8.9 months with cetuximab plus FOLFIRI and 8.0 months with FOLFIRI alone) for the first-line therapy of mCRC. The similar situation occurred with bevacizumab [19]. In E3200 study, the addition of bevacizumab to chemotherapy resulted in a statistically significant improvement in OS for patients with previously treated mCRC [20]. However, in No16966 trial, the addition of bevacizumab to chemotherapy could not prolong OS for the first-line treatment of mCRC [21]. Furthermore, in AVAGAST trial, the addition of bevacizumab to chemotherapy didn’t improve the OS for the first-line of MGC [22]. However, in RAINBOW study, the addition of ramucirumab, which has similar mechanism of action with bevacizumab, could increase median OS in second-line treatment for patients with MGC [6]. So, the failure of EGFR antagonists in the first-line setting of MGC in both EXPAND and REAL3 trials could not conclude that cetuximab was useless when combined with other drugs or in the second-line setting. In our study, preliminary exciting effects were obtained when cetuximab combined with irinotecan and 5-Fu civ in second-line setting, which deserves to be confirmed in further randomized controlled clinical trials.

Moreover, gastric cancer may comprise a group of heterogeneous diseases that differ in the expression of cell-signaling molecules and have varying degrees of metaplasia, and therapy in a molecularly selected population may result in better outcomes. Therefore, potential biomarkers of cetuximab therapy in combination with FOLFIRI as a second-line treatment in MGC patients were selected and analyzed based on their roles in EGFR-mediated signaling in our study. Mutations in KRAS, BRAF and PIK3CA genes were not identified. In accordance with previous reports, the frequency of KRAS activating mutations was found to be low in GC patients [23]. The efficacy of cetuximab is limited to patients with KRAS wild-type tumors in mCRC [24]. However, unlike in mCRC where KRAS mutation frequencies are approximately 35% to 45%, KRAS was not identified as a suitable predictive marker of cetuximab efficacy in GC [25, 26]. Protein expression analyses (pEGFR and pAKT expression) also had negative results in our study.

All grades of acne-like rash occurred in 70.8% of patients and grades 3/4 toxicities were observed in 9.8% of the patients, and this side-effect did not correlate with the clinical outcomes in this study. Although the associations of the presence and severity of cetuximab-related skin rash with clinical outcome have been reported in mCRC patients [27], but the role of cetuximab-related skin rash in clinical outcome remains inconclusive in AGC. In the FOLCETUX study, RR values were higher in patients with skin rash grade ≥2 compared with grade <2 (53% vs. 33%), but the difference was not statistically significant [11]. Similar results were reported by another study [28].

In gastric cancer, it has been reported that VEGF expression was associated with tumor aggressiveness and poor prognosis [29, 30]. Juttner S et al found that elevated circulating VEGF levels could promote tumor aggression and shorten survival in patients with gastric cancer [31]. Jung YD et al found that the inhibition of VEGFR-2 could decrease tumor growth and vascularization in animal models of gastric cancer [32]. Ramucirumab, a human IgG1 monoclonal antibody VEGFR-2 antagonist, has been proven to prolong OS in the second-line treatment of MGC either as monodrug or combined with paclitaxel. These results suggested VEGF and VEGFR-2-mediated signalling and angiogenesis contribute to the pathogenesis of gastric cancer. Vincenzi and colleagues revealed the reduction of serum VEGF levels could predict the efficacy of treatment with cetuximab plus irinotecan in heavily pretreated mCRC patients [33]. Therefore in this study we also annalyzed the value of VEGF as a potential marker, and our data showed patients with low baseline plasma VEGF levels experienced a more favorable outcomes. In patients with baseline plasma VEGF levels less than12.6 pg/ml, OS time was prolonged by up to 12 months compared with 5 months in patients with VEGF levels higher than 12.6 pg/ml (*P* <0.0001), so were the TTP (6.9 months vs. 2.8 months, respectively, *P* = 0.0005) and the RR (55.0% vs. 5.3%).

Our findings are consistent with recent studies suggesting that EGFR signaling pathways are involved in tumor angiogenesis, especially through the upregulation of VEGF. The phosphorylation of EGFR signalling could lead to the activation of PI3K/AKT and RAS/RAF/MEK/MAPK pathways, which could induce tumor angiogenesis. EGFR antagonists could inhibit angiogenic growth factor production (VEGF) and tumor-induced angiogenesis [34]. Khong et al found that EGFR phosphorylation activates the MAP kinase signalling and promotes HIF stabilisation in CRC. HIF activation and EGF-mediated signalling could induce the activation of angiogenic genes, such as ANGPTL4, EFNA3, TGFβ1 and VEGF [35]. It is hypothesized that elevated VEGF, which promotes tumor angiogenesis, induces acquired resistance to EGFR treatment. Grimminger et al found that pretreatment intratumoral VEGF mRNA expression levels are predictive markers of pathologic response to neoadjuvant cetuximab based chemoradiation in locally advanced rectal cancer [36]. Preclinical studies point out that inhibition of EGFR by cetuximab could downregulate the expression of VEGF [37, 38]. Viloria-Petit A et al reported that A431 cells with overexpression of VEGF were resistant to anti-EGFR antibodies and A431 xenografts with acquired resistance to anti-EGFR antibodies showed higher levels of VEGF [39]. Bianco R et al also found that GEO colon cancer cells with increased VEGF expression were resistant to EGFR inhibitors and VEGFR-1 tyrosine kinase inhibitor could reduce tumor growth in animal models [40]. These observations suggested that VEGF pathway plays an important role in mediating tumor responses and drug resistance to anti-EGFR therapies. The importance of VEGF pathway in MGC has recently been magnified by the positive results with Ramucurimab in MGC. However, the biomarker analyses are exploratory in nature in our study.

## Conclusions

In conclusion, our study showed cetuximab combined with mFOLFIRI was well tolerated and preliminary encouraging efficacy data were obtained in the second-line treatment of MGC. Furthermore, biomarker analysis indicated that gastric cancer patients with low baseline circulating VEGF levels have better clinical outcomes. As our study is single arm, the value of cetuximab in the second-line treatment of MGC and the value of biomarker need to be confirmed in further randomized controlled clinical trials.

## Abbreviations

BSC: Best supportive care
CR: Complete response
ECOG: Eastern cooperative oncology group
EGFR: Endothelial growth factor receptor
mCRC: Metastatic colorectal cancer
MGC: Metastatic gastric cancer
OS: Overall survival
PD: Progressive disease.
PFS: Progression free survival
PR: Partial response
PS: Performance status
SD: Stable disease
SLC: Salvage chemotherapy
TTP: Time-to-progression
VEGF: Vascular endothelial growth factor
